# Supramolecular assemblies from antimony(V) complexes for the treatment of leishmaniasis

**DOI:** 10.1007/s12551-023-01073-6

**Published:** 2023-06-06

**Authors:** Cynthia Demicheli, Virgínia M. R. Vallejos, Juliane S. Lanza, Guilherme S. Ramos, Bruno R. Do Prado, Sébastien Pomel, Philippe M. Loiseau, Frédéric Frézard

**Affiliations:** 1 Department of Chemistry, Institute of Exact Sciences, Universidade Federal de Minas Gerais, Belo Horizonte, MG 31270-901 Brazil; 2 Department of Physiology and Biophysics, Institute of Biological Sciences, Universidade Federal de Minas Gerais, Belo Horizonte, MG 31270-901 Brazil; 3 Vaxinano, 59120 Loos, France; 4 Faculty of Pharmacy, Antiparasite Chemotherapy (PARACHEM), UMR 8076 CNRS BioCIS, University Paris-Saclay, 91400 Orsay, France

**Keywords:** Antimony, Leishmaniasis, Nanoassemblies, Supramolecular, Pnictogen bonding, Drug delivery

## Abstract

The pentavalent meglumine antimoniate (MA) is still a first-line drug in the treatment of leishmaniasis in several countries. As an attempt to elucidate its mechanism of action and develop new antimonial drugs with improved therapeutic profile, Sb(V) complexes with different ligands, including β-cyclodextrin (β-CD), nucleosides and non-ionic surfactants, have been studied. Interestingly, Sb(V) oxide, MA, its complex with β-CD, Sb(V)-guanosine complex and amphiphilic Sb(V) complexes with N-alkyl-N-methylglucamide, have shown marked tendency to self-assemble in aqueous solutions, forming nanoaggregates, hydrogel or micelle-like nanoparticles. Surprisingly, the resulting assemblies presented in most cases slow dissociation kinetics upon dilution and a strong influence of pH, which impacted on their pharmacokinetic and therapeutic properties against leishmaniasis. To explain this unique property, we raised the hypothesis that multiple pnictogen bonds could contribute to the formation of these assemblies and their kinetic of dissociation. The present article reviews our current knowledge on the structural organization and physicochemical characteristics of Sb-based supramolecular assemblies, as well as their pharmacological properties and potential for treatment of leishmaniasis. This review supports the feasibility of the rational design of new Sb(V) complexes with supramolecular assemblies for the safe and effective treatment of leishmaniasis.

## Introduction

Pentavalent antimonials, including meglumine antimoniate (MA) (Glucantime®, Sanofi-Aventis Pharmaceutical Ltda, São Paulo, Brazil) and sodium stibogluconate (Pentostam®), are still used in developing countries for the treatment of leishmaniasis (Sundar and Chakravarty 2015; WHO 2020; PAHS 2022). This disease is caused by protozoan parasites of the genus *Leishmania*, which are transmitted by the bites of Phlebotomine sandflies. The *Leishmania* parasite is a motile promastigote in the sandfly, that transforms into a round non-flagellar amastigote inside the host macrophage, and resides in the acidic environment of secondary lysosomes. Distinct species of *Leishmania* cause different clinical manifestations, including cutaneous (CL), mucocutaneous (MC) and visceral leishmaniasis (VL). Leishmaniasis is classified as a neglected tropical disease, as it affects predominantly poor populations of developing countries. There are few effective drugs available for the treatment of the disease and limited public and private commitments for new drug development (WHO 2020).

Depending on the clinical form of leishmaniasis, Glucantime® is given by either intralesional, intramuscular or intravenous route, with repeated injections for a long period of time (OPAS 2022). Antimonial treatment often leads to systemic side effects, such as nausea, myalgia, diarrhea, skin rashes, renal and hepatotoxicity, together with severe pancreatitis and cardiotoxicity. All these factors decrease patient compliance and contribute to treatment failure (Marsden 1985; Berman 1997). Consequently, antimony-resistant *Leishmania* strains have emerged, worsening the scenario of leishmaniasis therapy (Ponte-sucre et al. 2017).

Despite the introduction of pentavalent antimonial drugs in the 1940s, progress towards the understanding of the chemistry of Sb(V) and their mechanism of action has been slow, and some fundamental aspects are still not completely understood (Frézard et al. 2009). The structural characterization of inorganic Sb(V) complexes has been especially difficult due to their tendency to form oligomers and amorphous state. Today, the most well-accepted model for the mode of action of pentavalent antimonials is that Sb(V) behaves as a prodrug that is activated through reduction into the more active and toxic trivalent form (Goodwin and Page 1943; Ferreira et al. 2003; Zhou et al. 2004). Then, Sb(III) generates oxidative stress in the *Leishmania* cell through binding to trypanothione and trypanothione reductase (TR) (Yan et al. 2003; Baiocco et al. 2009) with consequent interference in the thiol metabolism (Wyllie et al. 2004). But there is evidence that Sb(V) by itself may also exert pharmacological actions, for instance through complexation with ribonucleosides and modulation of the host immune response (Pathak and Yi 2001; Demicheli et al. 2002; Frézard et al. 2009). Typical changes observed in Sb-resistant *Leishmania* strains encompass increased levels of intracellular thiols, overexpression of ATP-binding cassette (ABC) transporters responsible for Sb(III) sequestration inside intracellular vesicles or active extrusion out of the cells and down-regulation of the aquaglyceroporin (AQP1) that promotes Sb(III) entry into the parasite (Mukhopadhyay et al. 1996; Légaré et al. 2001; Gourbal et al. 2004; Frézard et al. 2014). Resistance to Sb may also be mediated by the host cell due to upregulation of ABC transporters, resulting in a nonaccumulation of intracellular Sb(III) (Mookerjee et al. 2008).

Besides the gaps of knowledge regarding the chemistry and biochemistry of pentavalent antimonials, the status of leishmaniasis as a neglected tropical disease has contributed to hamper the discovery of more effective and less toxic pentavalent antimonial drugs. In this context, the development of orally and topically active drugs is especially important, as the disease often affects people with limited access to health centers.

Supramolecular chemistry has been the subject of intense research since the 1980s, due to its relevance for the understanding of the fundamental principles of host–guest interactions and their innumerous applications (Lehn 1988). It encompasses the critical role of noncovalent interactions in the structure and function of supramolecular assemblies. Typical application in biology refers to elucidation of the mechanisms involved in substrate—receptor recognition. In medicine, the unique and often advantageous properties of supramolecular assemblies have led to exploration of their use for drug delivery, disease diagnosis and imaging, and regenerative medicine (Webber and Langer 2017; Hou et al. 2022). In this context, the emergence of nanostructure-based drug delivery systems has provided new tools for optimizing the pharmacokinetic profile of drugs and their therapeutic efficacy. The concept of self-assembling prodrug was also recently introduced to expand the area of conventional prodrug design, as a new pathway towards more effective therapies (Cheetham et al. 2017). The dynamic nature of metal − ligand coordination has also been explored to achieve metallo-organic supramolecules and control their architecture and function (Northrop et al. 2009).

The aims of the present review are to describe the supramolecular assemblies formed by different antimonial compounds in aqueous solutions and to report the impact of the supramolecular organization on their pharmacological properties and potential benefits against leishmaniasis. Emphasis is given on the unique chemical features of Sb(V).

## Antimony(V) complexes forming supramolecular assemblies

We present below different Sb(V) complexes that were found to self-associate into supramolecular assemblies and were evaluated for their pharmacokinetic properties and antileishmanial activity.

As described in detail below and summarized in Table 1, different types of nanoassemblies were observed and the supramolecular organization was found to impact their pharmacokinetics and therapeutic activities.

**Table 1:** Supramolecular assemblies obtained from antimony(V) complexes as drug delivery systems against experimental leishmaniasis

Complex	Type of supramolecular assembly	Physicochemical characteristics	Main pharmacokinetic / therapeutic impact	References
(NMG–Sb)_*n*_-NMG	Nanoaggregates in aqueous media	Slow dissociation upon dilution in water	Reduced cytotoxicity; high hepatic accumulation; low oral bioavailability	Carter et al. 1988; Roberts et al. 1998; Frézard et al. 2008a; Dzamitika et al. 2006; Ferreira et al. 2010; Ribeiro et al. 2010
(NMG-Sb)_*n*_-β-CD	Nanoaggregates in aqueous media	Slow dissociation upon dilution in water	Improved oral bioavailability and oral efficacy in murine CL	Demicheli et al. 2004; Martins et al. 2006; Frézard et al. 2008b
Sb-G and Sb(G)_2_	Hydrogel	Formation of nanoparticles upon dilution in water	High in vitro antileishmanial activity	Demicheli et al. 2006; Ferreira et al. 2010
Sb(L8)_3_ Sb(L10)_3_	Micelle-like and higher order nanoaggregates In aqueous media	Formation of hydrophobic environment with greater kinetic stability at neutral pH than at acidic pH	Antileishmanial efficacy by oral route in murine VL and CL; targeting the liver, macrophages and intracellular parasite after parenteral administration	Fernandes et al. 2013; Lanza et al. 2016, 2022

NMG: N-methyl-D-glucamine; G: guanosine; L8: N-octanoyl-N-methylglucamide; L10: N-decanoyl-N-methylglucamine

### Meglumine antimoniate

#### Physicochemical characterization and evidence for supramolecular nanoassemblies

**Fig. 1:**
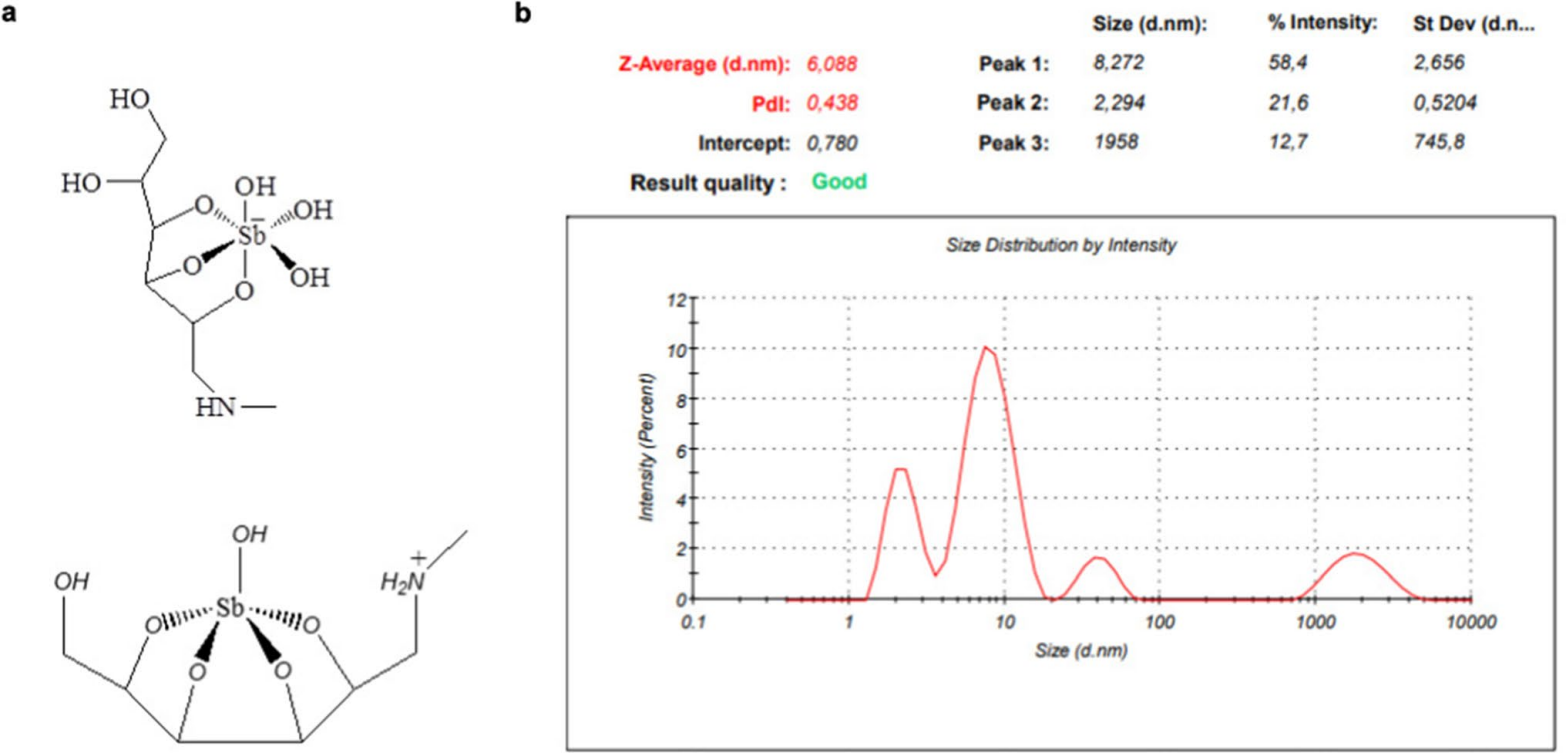
Sb(V) species identified by ESI–MS in the positive and the negative modes in diluted aqueous solution of MA (Frézard et al. 2008a) (**a**) and evidence by DLS for existence of supramolecular assemblies in Glucantime® (**b**)

MA can be synthesized from the reaction of N-methyl-D-glucamine (NMG) with Sb(V) oxide, obtained either from the hydrolysis of SbCl_5_ in water or from an antimonial salt, like KSb(OH)_6_ (Frézard et al. 2009). Analyses of MA by electrospray ionization mass spectrometry (ESI–MS) in the positive and negative modes have indicated that it is composed of a mixture of oligomeric structures with the general formula (NMG–Sb)_*n*_-NMG and (NMG–Sb)_*n*_ (Frézard et al. 2008a). As illustrated in Fig. 1a, these species can be cationic, anionic or zwitterionic, depending on the ionization states of antimoniate or amino groups. Characterization of Glucantime® through osmolarity measurement and C, H, N, Na, Cl^−^ and Sb analyses has suggested the predominance of 2:3 Sb-NMG complex in the concentrated liquid formulation (Kato et al. 2018).

Upon dilution of the original drug solution to 0.1 M of Sb, a slow decrease of the osmolarity was reported with a half-time of 20 min at 37 °C, indicating slow dissociation to 1:1 Sb-NMG complex (Roberts et al. 1998; Frézard et al. 2008a) (Fig. 1a). The increase of temperature also favored its dissociation (Martins et al. 2006).

Evidence for the formation of supramolecular assemblies in Glucantime® was obtained by dynamic light scattering (DLS) analysis (Ribeiro et al. 2010). As illustrated in Fig. 1b, polydisperse nanoparticles, with mean hydrodynamic diameter of about 6 nm and polydispersity index of 0.42, were identified. This may explain why the ultrafiltration of Glucantime® through a 3 kDa (MWCO) cellulose membrane was less efficient than that of similar, less polymerized forms of MA (Kato et al. 2018).

The formation of high-order complexes probably arises from oligomerization through formation of multiple Sb(-O-C)_*n*_ covalent bonds. But it is also likely that larger nanoaggregates are stabilized by noncovalent interactions such as hydrogen bonding, as well as electrostatic and dipole − dipole interactions.

#### Impact of the supramolecular organization on the pharmacological properties

The complexation of KSb(OH)_6_ with NMG was found to markedly reduce its cytotoxicity (Dzamitika et al. 2006; Ferreira et al. 2010), suggesting that MA acts as a slow release system of Sb(V).

The formation of nanoassemblies in Glucantime® certainly influences the drug pharmacokinetics and distribution after parenteral administration. The nanoparticulate composition probably explains the high drug accumulation in the liver following intravenous administration (Carter et al. 1988) and the hepatotoxic effect associated to antimonial treatment (Kato et al. 2014). Indeed, Kupffer cells in the liver are known to remove nanoparticles and other colloidal from the blood circulation through a filter effect.

The highly polymerized state of MA in Glucantime® also resulted in its reduced antileishmanial activity by oral route in a murine model of VL, when compared to less polymerized forms of MA (Kato et al. 2018). The lower activity of Glucantime® given orally also correlated with a lower cellular uptake and hepatic accumulation of Sb.

### Meglumine antimoniate-β-cyclodextrin complex

#### Physicochemical characterization and evidence for supramolecular nanoassemblies

**Fig. 2:**
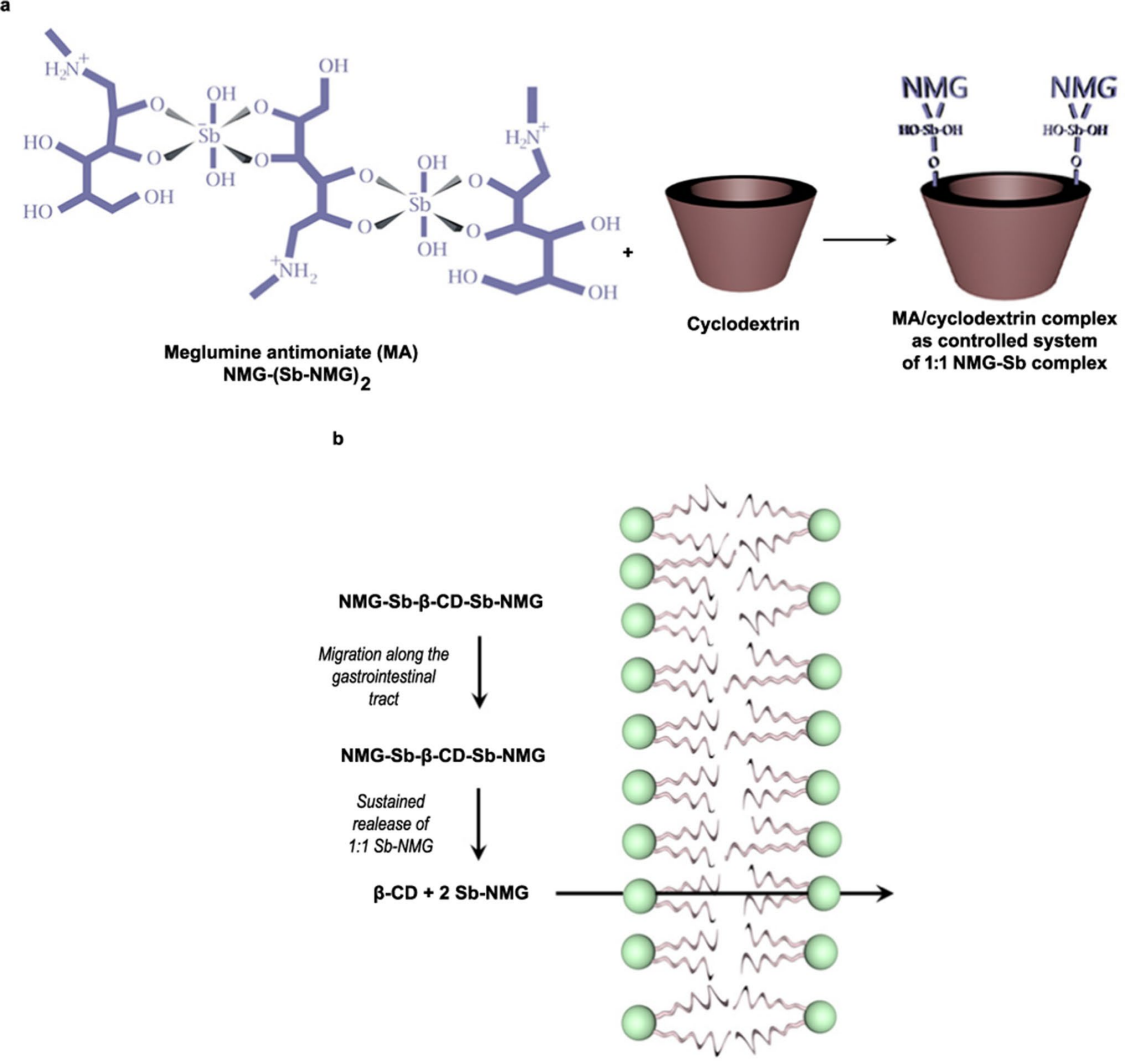
Proposed mode of action of MA-β-CD ternary complex to increase the serum level of Sb after oral administration. The MA/β-CD composition consists in high molecular-weight ternary complexes, such as NMG-Sb-β-CD-Sb-NMG species, which migrate along the gastrointestinal tract and slowly release MA in the form of 1:1 Sb-NMG complex that permeates by simple diffusion across the intestinal epithelium. Figure 2b is reprinted from Frézard et al. (2008b) and minimally adapted, with permission from Elsevier

β-cyclodextrin (β-CD) has been investigated as a potential carrier for MA. Cyclodextrins (CDs) are cyclic oligosaccharides composed of glucose units joined through α-1.4 glycosidic bonds. They are formed from 6, 7 and 8 units of glucose, known as α-CD, β-CD and γ-CD, respectively. CDs display a torus-like cone shape (Fig. 2b), in which the secondary hydroxyl groups can be found at the broadest end, bonded to the C2 and C3 atoms of the glucose units, while the primary hydroxyl groups are located at the narrower opposite end (Hirayama and Uekama 1999). Of all natural CDs, β-CD has the lowest solubility, due to the high number of intramolecular hydrogen bonds among secondary hydroxyl groups within the molecule. The CD truncated cone exhibits an external hydrophilic layer due to the hydroxyls and an internal hydrophobic cavity. CDs are well known in recognition chemistry as molecular hosts capable of including, with a degree of selectivity, water-insoluble guest molecules by means of noncovalent interactions within their hydrophobic cavity. This has led to their use as carriers of poorly water-soluble drugs, by exploiting the enhancement of the drug apparent solubility and dissolution rate. CDs are generally regarded as safe when given by the oral route, as they exhibit very low membrane permeability and minimal intestinal absorption. On the other hand, α-CD and β-CD are not recommended for parenteral administration, because they show nephrotoxicity and hemolytic activity (Irie and Uekama 1997). To reduce the toxicity of CDs and improve their solubility and inclusion capacity, chemical modifications into the primary and secondary hydroxyl groups have been introduced, such as substitution with hydroxypropyl or sulfobutyl (Rajewski and Stella 1996).

As an attempt to improve the oral bioavailability of MA by the oral route, 1:1 MA-β-CD complex was prepared in two steps: first, the reaction of MA with β-CD through heating of aqueous solution at 55 °C; secondly, the freeze-drying of the resulting solution (Demicheli et al. 2004). The first step was found to promote the depolymerization of MA into 1:1 NMG-Sb complex and the formation of ternary 1:1:1 and 2:2:1 NMG-Sb-β-CD complexes (Martins et al. 2006). The second step promoted additional interactions and the formation of supramolecular nanoassemblies, as evidenced by circular dichroism, DLS and ESI(-)-MS analyses (Frézard et al. 2008b). These nanoassemblies slowly dissociated upon dilution in water, according to circular dichroism, DLS and ^1^H NMR characterization, supporting their ability to act as a sustained drug release system. Again, it is likely that noncovalent interactions such as hydrogen bonding (involving hydroxyl and amino groups) electrostatic and dipole − dipole interactions contributed to the stability of the larger nanoaggregates.

#### Impact of the supramolecular organization on the pharmacological properties

The MA-β-CD ternary complex showed greater antileishmanial activity than MA when given by the oral route in murine model of CL (Demicheli et al. 2004). Interestingly, the MA/β-CD composition differed markedly from conventional inclusion complexes between cyclodextrins and poorly water-soluble drugs, as it involved covalent and noncovalent bonds, but no inclusion of part of the guest molecule inside the hydrophobic cavity of the cyclodextrin. In the study of the mode of action of the non-conventional drug-cyclodextrin complex, both the depolymerization of MA and the supramolecular organization were found to contribute to higher serum levels of Sb, following oral administration in mice (Martins et al. 2006; Frézard et al. 2008b). The proposed mode of action is illustrated in Fig. 2.

Translation of the new oral composition from the rodent model to dogs or humans was hampered by the limited solubility of β-CD and the need to administer large volumes and high amount of β-CD to achieve therapeutic dose of Sb.

Another potential application of MA-β-CD ternary complex refers to treatment of CL by topical application to the skin lesion. This proposal is supported by the therapeutic efficacy of intralesional MA in CL (Carvalho et al. 2019). Topical treatment would present the advantage of higher comfort and compliance of the patient, when compared to intralesional injection. Comparison of the percutaneous absorption of Sb in Franz diffusion cell using mouse skin with or without stratum corneum indicated a higher permeation rate from MA-β-CD than MA alone (Martins et al. 2006). However, the proof of concept of the therapeutic efficacy of a topical formulation of MA-CD against CL is still pending.

### Sb(V)-guanosine complex

#### Physicochemical characterization and supramolecular assemblies

In the search of a molecular target for Sb(V), Demicheli et al. (2002) discovered that KSb(OH)_6_ formed stable complexes with ribonucleosides. The formation of 1:1 and 1:2 Sb(V)-ribonucleoside complexes from adenosine (A), guanosine (G), cytosine (C) and uridine (U) was evidenced by NMR, mass spectrometry and circular dichroism analyses (Demicheli et al. 2002, 2006; Chai et al. 2005; Ferreira et al. 2010). The changes in H2′ NMR resonance supported the binding of Sb(V) to the ribose hydroxyl groups, via ring chelation at C2′ and C3′. Interestingly, 1:1 and 1:2 Sb(V)-GMP complexes were found to be kinetically stable upon dilution at neutral pH, but to rapidly dissociate at pH 5 (Ferreira et al. 2006). Upon dilution in water at pH 5 and incubation at 37 °C, the 1:1 Sb(V)-GMP complex dissociated with a half-time of about 2.8 h. As faster dissociation was expected in more acidic media such as the gastric fluid, these complexes were not investigated for oral administration.

Of special interest, regarding the formation of supramolecular assemblies, is the complexation of Sb(V) with G. Following reaction of KSb(OH)_6_ with G in water at 60 °C (Sb(V)/G molar ratio varying from 0.5 to 1) and cooling of the resulting solution at room temperature, a translucent thermoreversible hydrogel was formed (Demicheli et al. 2006). After freeze-drying and reconstitution with water, the Sb(V)/G mixture recovered as a hydrogel. This is in contrast with G alone that solubilized in water upon heating but precipitated upon cooling at room temperature.

**Fig. 3:**
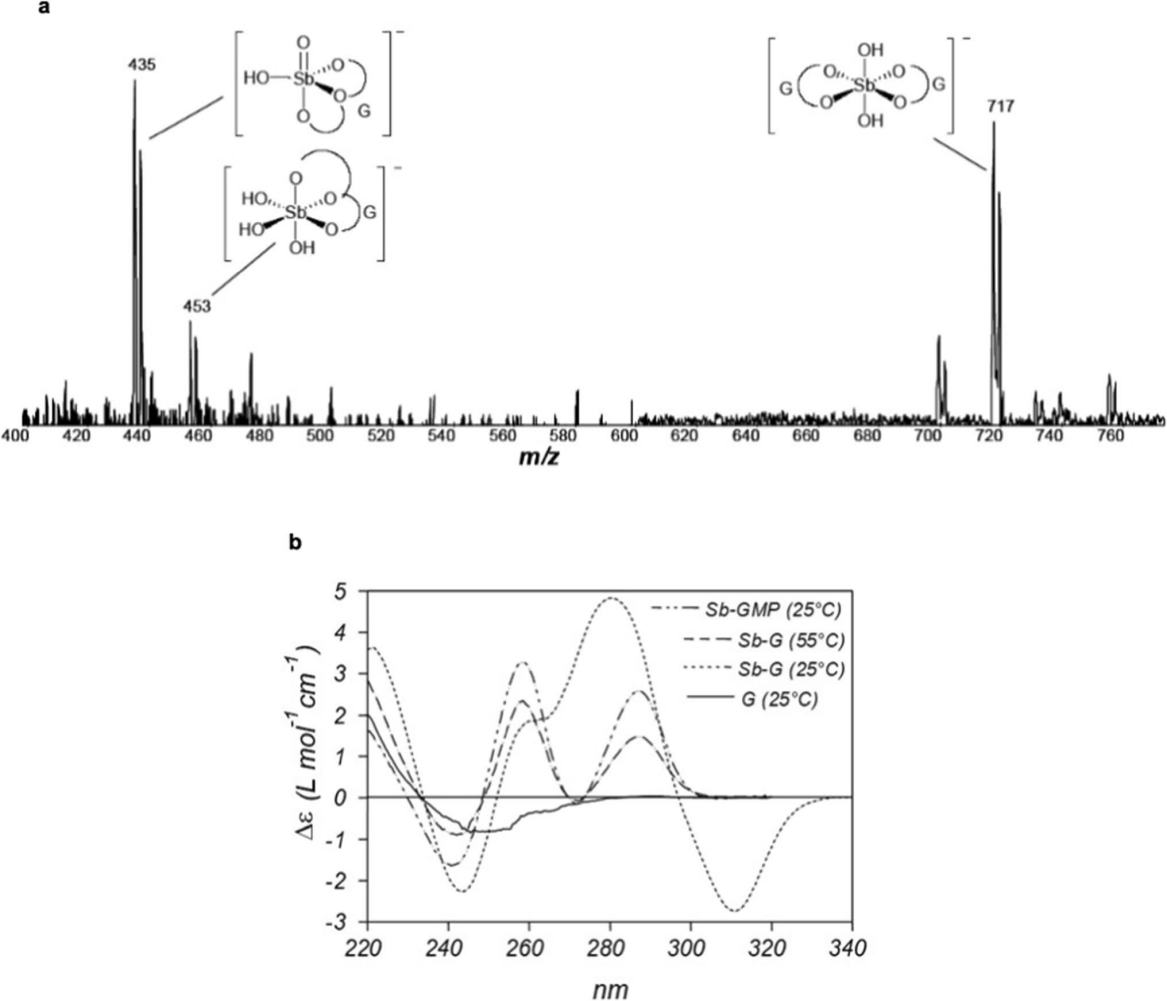
1:1 and 1:2 Sb(V)-G complexes identified by ESI–MS (**a**) and characterization of the supramolecular assemblies by circular dichroism (**b**). (**a**) ESI–MS(-) spectrum of the Sb(V)–G hydrogel, registered after dilution in water/methanol. (**b**) Circular dichroism spectra obtained for G and Sb(V)-G mixture at 1:2 molar ratio, just after heating for 90 min at 60 °C and after cooling at room temperature and for Sb(V)-GMP mixture (1:2 molar ratio) after heating and cooling. Samples were diluted in water at 1 mM of G for measurement. Figures 3a and 3b reprinted from Demicheli et al. (2006) with permission from Elsevier

ESI–MS spectra showed the co-existence of 1:2 and 1:1 Sb(V)–G species in the hydrogel, as illustrated in Fig. 3a. NMR analysis indicated that the gel consisted in 74% 1:1 Sb(V)–G complex, 20% 1:2 Sb(V)–G complex and 6% free G.

Circular dichroism analysis was also performed to further characterize the supramolecular organization of the hydrogel. Figure 3b shows the circular dichroism spectra obtained for Sb(V)–G mixture at a 1:2 molar ratio after heating for 1.5 h at 60 °C, cooling at room temperature and subsequent dilution in water for measurement. Comparison was made with free G and the Sb(V)–GMP mixture at a 1:2 molar ratio after heating and cooling at room temperature with no gel formation.

The spectrum of the Sb(V)–G mixture after heating (before gel formation) was similar to that of the Sb(V)–GMP mixture. However, this spectrum differed markedly from that of free G, evidencing Sb(V) complexation. A couplet-type signal centered at 248 nm with two Cotton effects of opposite signs, a negative Cotton effect at 241 nm and a positive Cotton effect at 258 nm, was observed. Such a signal can be attributed to excitonic coupling between nucleobases that occurs in the 1:2 Sb(V)–G complex.

Cooling of the Sb(V)–G mixture induced additional changes in the spectrum, with the appearance of an intense couplet-type signal centered at 297 nm with a positive Cotton effect at 280 nm and a negative Cotton effect at 311 nm. The new signal is probably due to additional interactions between nucleobases involving excitonic coupling, most probably base stacking, also supported by a 30% hypochromic effect. At this second organization level, a left-handed screw conformation exists between the transition dipole moments; i.e., the chirality is negative.

According to this data, the Sb(V)–G hydrogel results from two types of interactions: (i) Sb-O-C covalent bonds in Sb(V)–G complexes and (ii) intermolecular interactions between the different Sb(V)–G complexes, including base stacking. The G quartets formed by the self-assembly of four guanine bases, as described previously in guanine-rich oligonucleotides (Guschlbauer et al. 1990), may also contribute to the supramolecular architecture.

#### Potential for topical treatment of CL

Among Sb(V) complexes with the ribonucleosides U, C, A and G, Sb(V)-G displayed the greatest antileishmanial activity in the intramacrophage *Leishmania* amastigote model (Ferreira et al. 2010). Nanoparticles with 10-nm diameter, which are formed after dilution of Sb(V)-G gel in aqueous media (Demicheli et al. 2006), likely suffered internalization by the macrophages, resulting in enhanced drug delivery to the intracellular parasite.

The Sb-G hydrogel has potential in the treatment of CL by topical application to the skin lesion. Considering the strong pH dependence of the stability of Sb-G complexes, the pH of the skin appears to be a critical parameter for the release of Sb-G complexes and Sb(V) from the gel. The pH of the stratum corneum of healthy skin is 4.1–5.8, however, the pH was found to increase in lesioned skin (Proksch 2018; Frick et al. 2022). Thus, in healthy intact skin, one would expect a fast release of Sb(V) at the gel-skin interface. On the other hand, a more sustained release of Sb(V) should occur in the lesioned skin. Therefore, it would interesting to study in the future the therapeutic efficacy of Sb-G hydrogel in the murine model of CL.

### Sb(V) complexes with non-ionic surfactants

#### Physicochemical characterization and supramolecular nanoassemblies

**Fig. 4:**
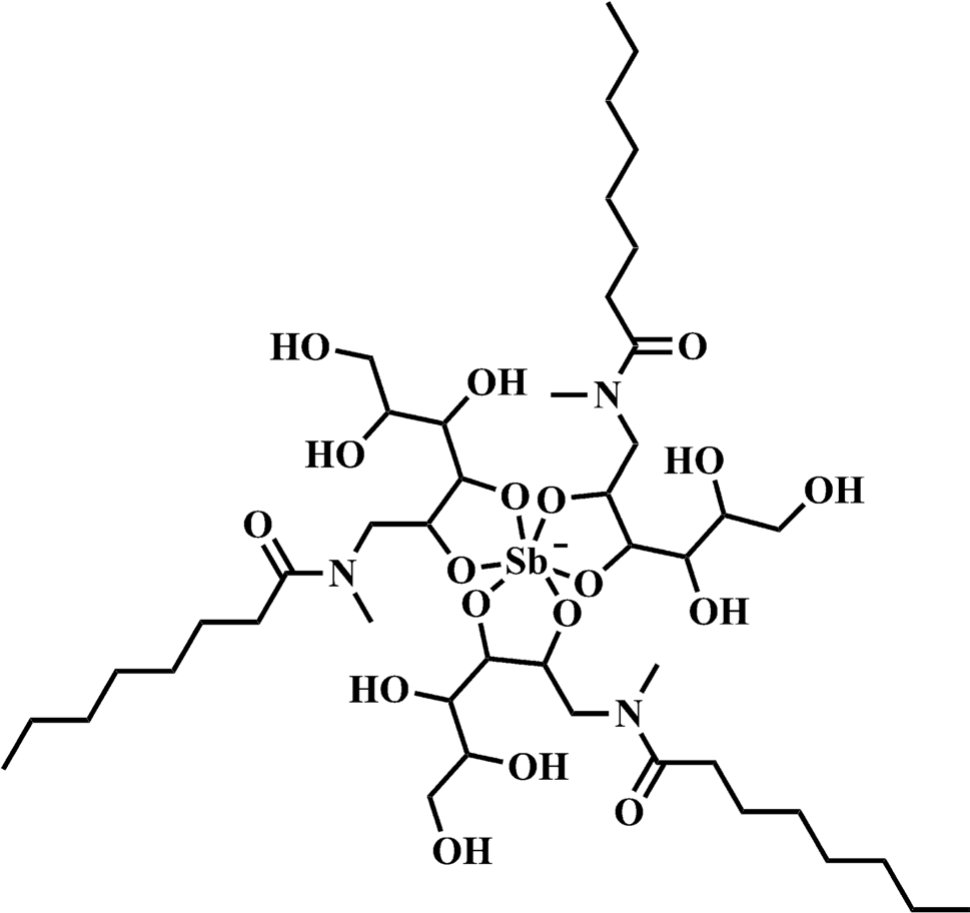
Structure of the main species identified in SbL8 by ESI–MS. Figure reprinted from Lanza et al. (2022), in accordance with Creative Commons Attribution 4.0 International

Amphiphilic Sb(V) complexes were first introduced to achieve orally effective pentavalent antimonials (Fernandes et al. 2013). This strategy was supported by previous works evidencing enhanced permeation of metal ions across biological membranes, through their complexation with lipophilic ligand, as, for example, in 99Tcm-hexamethylpropyleneamine oxime (Ell et al. 1985) and satraplatin (Neidle et al. 1995). Two complexes, named SbL8 and SbL10, were obtained through reaction of KSb(OH)_6_ at 1:3 molar ratio with the non-ionic surfactants N-octanoyl-N-methylglucamide (L8) and N-decanoyl-N-methylglucamine (L10), respectively. N-alkyl-N-methylglucamides were chosen as ligands, because their polar head moiety is NMG, which is the ligand of Sb(V) in MA. Comparative ^13^C NMR analyses of L8 and its SbL8 supported the complexation of Sb(V) with the polar head of the surfactant. According to ^1^H NMR analyses in d_6_-DMSO, integration of the proton signals related to hydroxyls indicated that Sb(V) coordinated between 2 and 3 hydroxyls in L8, supporting the formation of 1:2 and 1:3 Sb-L complexes (Fernandes et al. 2013). ESI–MS(-) analysis of SbL8 and SbL10 evidenced a predominant peak corresponding to 1:3 Sb-L complexes and lower-intensity peaks related to 1:2 and 2:5 Sb-L species. Figure 4 displays the structural formula proposed for the 1:3 species identified in SbL8.

**Fig. 5:**
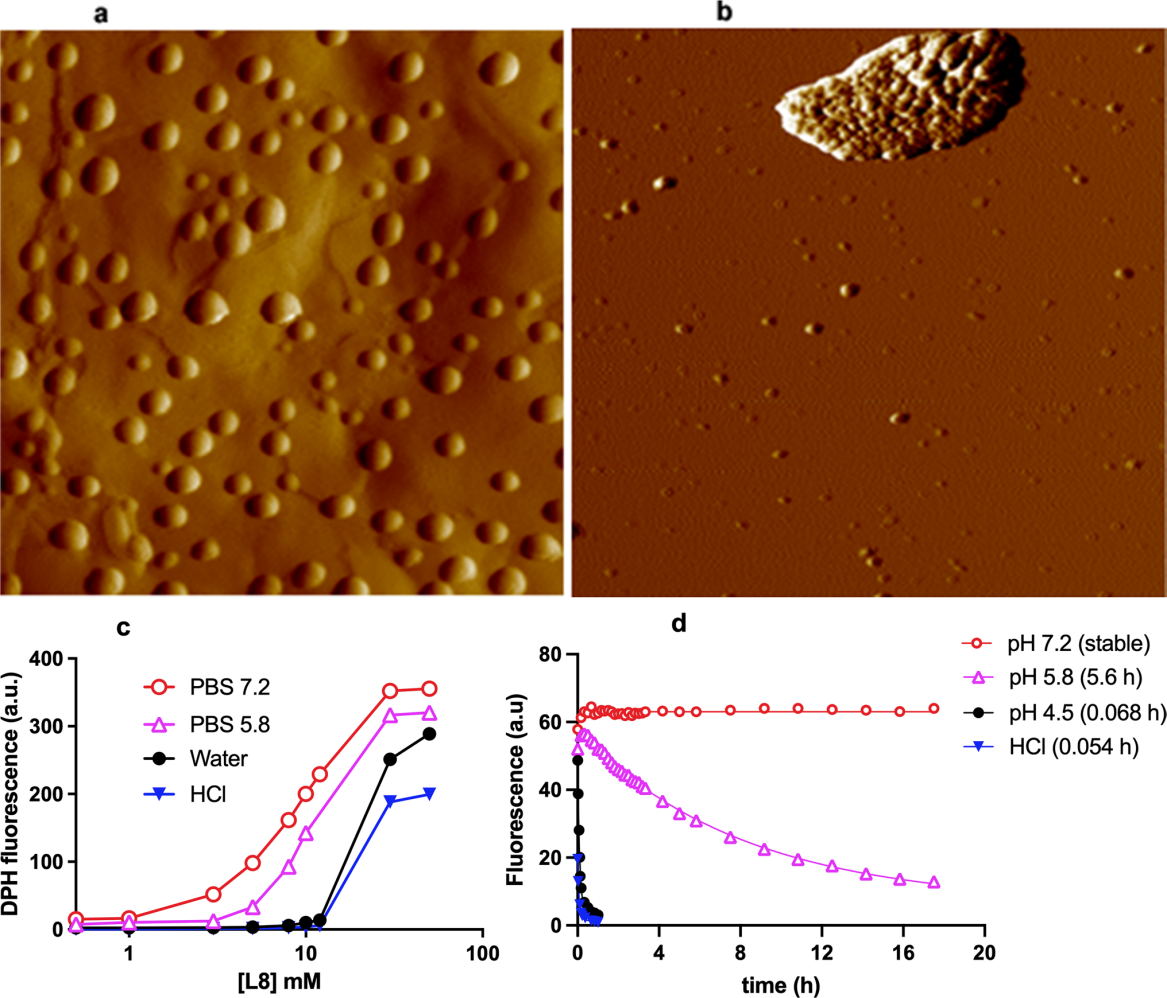
Characterization of the supramolecular assemblies in SbL8, using atomic force microscopy (AFM) (**a**, **b**) and diphenyhexatriene (DPH) fluorescence at equilibrium (**c**) and after dilution (**d**). Amplitude AFM images (2 µm) of SbL8 in (**a**) propylene glycol and (**b**) PBS 7.2, registered just after deposition and partial drying onto hydrophobic parafilm and cleaved mica, respectively. DPH fluorescence as a function of L8 concentration in SbL8 suspensions in PBS either at pH 5.8 or 7.2, water (pH 5.5), and HCl 0.05 M after incubation for 24 h at 25 °C (**c**) or after dilution of DPH-loaded SbL8 suspensions at 37 °C in PBS at pH 7.2, 5.8 or 4.5 or HCl 0.05 M, and final L8 concentration of 1 mM (**d**). In (**d**), data were fitted according to mono-exponential decay (half-times of DPH release at different pHs are shown in brackets). Figures 5c and 5d were reprinted and minimally adapted from Lanza et al. (2022), in accordance with Creative Commons Attribution 4.0 International

SbL8 and SbL10 were found to self-assemble in aqueous solutions, forming nanosystems capable of incorporating lipophilic or amphiphilic substances, including diphenylhexatriene (DPH), miltefosine and amphotericin B (Fernandes et al. 2013; Lanza et al. 2016, 2022; Carregal et al. 2019). The characterization of SbL8 nanoassemblies in water by dynamic light scattering (DLS), nanoparticle tracking analysis (NTA), transmission electron microcopy (TEM), atomic force microscopy (AFM) and small-angle X-ray scattering (SAXS) supported the formation of anionic micelle-like nanosystems with a spherical core–shell, in addition to higher order aggregates (Fernandes et al. 2013; Lanza et al. 2016). Figure 5a and 5b show typical AFM images of the nanostructures formed by SbL8 in propylene glycol and PBS 7.2, respectively.

Probing the hydrophobic environment with the DPH fluorescent marker showed that hydrophobic environment is formed at much lower concentration for SbL8 (CMC = 10 mM) when compared to L8 (CMC = 60 mM) (Fernandes et al. 2013).

Interestingly, these nanoassemblies suffered conformational changes upon modification of the medium polarity or the pH (Lanza et al. 2016; 2022). Thus, the hydrophobic environment of SbL8 aqueous dispersion was lost when moving from water to 1:1 (v/v) propylene glycol / water, although nanoassemblies were still present according to TEM, SAXS and AFM analyses.

The pH also markedly affected the formation of hydrophobic environment in SbL8 solution, as illustrated in Fig. 5c. According to the partition of DPH probe, the neutral pH (7.2) favored the formation of hydrophobic compartments, compared to acidic pH (5.8). Increasing the ionic strength also seems to favor the appearance of hydrophobic nucleus, as indicated by comparison of DPH partitions between water and PBS (5.8). The lower stability of the nanoassemblies at acidic pH may be attributed to the acid–base properties of antimoniate and, more specifically, the protonation of the oxygen linked to Sb that may favor transformation of 1:3 Sb-ligand complexes into 1:2 Sb-ligand species.

Interestingly, the loss of the hydrophobic nucleus of the nanoassemblies upon dilution below the CMC was found to be a slow process strongly influenced by the pH of the medium, as illustrated for SbL8 in Fig. 5d. Dilution of SbL8 and SbL10 in PBS at neutral pH did not result in significant change in DPH fluorescence over 18 h. However, at pH 4.5, the conformation change was observed with half-times of 4 min and 43 min for SbL8 and SbL10, respectively. The influence of pH on the kinetic of loss of the hydrophobic nucleus led us to propose the use of these nanoassemblies as carriers of lipophilic drugs, capable of releasing them specifically in the acidic compartment of macrophages in which the *Leishmania* resides. The pH-dependence of the kinetic stability of SbL8 and SbL10 nanoassemblies correlates well with that of GMP-Sb(V) complexes (Ferreira et al. 2006), and may be attributed to the acid–base properties of both antimoniate and hydroxyl groups.

#### Impact of the supramolecular organization on oral delivery

When given to mice by the oral route, SbL8 aqueous suspension promoted significantly higher levels of Sb in the serum and liver, in comparison to oral Glucantime® (Fernandes et al. 2013). As a result, SbL8 showed antileishmanial activity following oral treatment of mice with VL (at 200 mg/kg/day for 20 days), with parasite suppression higher than 99.9% in the liver and spleen. This is in contrast with Glucantime® that did not significantly reduce the parasite load under the same treatment regimen.

The ability of amphiphilic Sb(V) complexes to solubilize in both water and non-polar solvents probably contributes to their effective permeation across biological membranes and high rate of intestinal absorption.

It is not clear yet whether the supramolecular architecture of the nanoparticles contributed positively to the improved oral absorption of Sb, since SbL8 and SbL10 nanoassemblies are expected to suffer conformational change upon exposition to the highly acidic gastric fluid environment and the impact of this change requires further investigation. An interesting observation is that SbL8 given orally to mice in a 1:1 (v/v) water:propylene glycol vehicle promoted significantly higher and more sustained levels of Sb in the serum and showed greater antileishmanial activity against CL, when compared to SbL8 in water (Lanza et al. 2016). Thus, the likely “more open” conformation adopted by SbL8 nanoassemblies in the less polar vehicle, with no hydrophobic nuclei, seems to favor the drug absorption by the oral route.

On the other hand, the conformational change of SbL8 and SbL10 nanoparticles at acidic pH indicates that they would release encapsulated lipophilic drugs in the stomach and would not be able to carry them until the intestine.

#### Impact of the supramolecular organization on topical delivery

SbL8 and SbL10 have been incorporated at 12% (w/v) Sb in 1:1 (v/v) water:propylene glycol vehicle containing 1% (w/v) hydroxyethyl cellulose. When evaluated as topical formulations in the treatment of murine CL, through daily application, a moderate antileishmanial activity was observed (Lanza et al. 2022). Thus, these formulations deserve further evaluation, for instance by changing the daily regimen into more usual twice-a-day application and by incorporating an additional antileishmanial agent.

A study of the Sb absorption across intact mouse skin in Franz diffusion cell has also shown that Sb permeated much more slowly from SbL8 than MA (Almeida 2012), suggesting that the supramolecular assembling may reduce the rate of Sb permeation from SbL8. Permeation may also be limited by the high affinity of amphiphilic Sb complexes for lipidic membranes resulting in low skin penetration. On the other hand, when using the mouse skin without stratum corneum (stripped skin) to simulate the barrier loss as frequently observed in CL when lesions progress to ulcers, SbL8 showed significant permeation of Sb and a more sustained drug release profile in comparison to MA. The SbL8 profile is expected to be favorable for the topical treatment of CL, as it would guarantee effective drug levels in the lesion for a longer period.

#### Impact of the supramolecular organization on delivery by parenteral route

The potential of SbL8 and SbL10 nanoassemblies for the delivery of Sb to the infection sites by parenteral route has been recently investigated (Lanza et al. 2022). THP-1 macrophage cells were found to accumulate 20 times larger amount of Sb from SbL8 and SbL10, compared to Glucantime®, after 4-h exposition at 37 °C. Such a high efficiency of Sb delivery to cells from the amphiphilic complexes may be attributed to their binding onto the cell surface, because of incorporation of their acyl chain into the lipid membrane. The negatively charged nanoparticles may also bind to macrophage scavenger receptors and be internalized by phagocytosis, as described for anionic liposomes (Tempone et al. 2004).

**Fig. 6:**
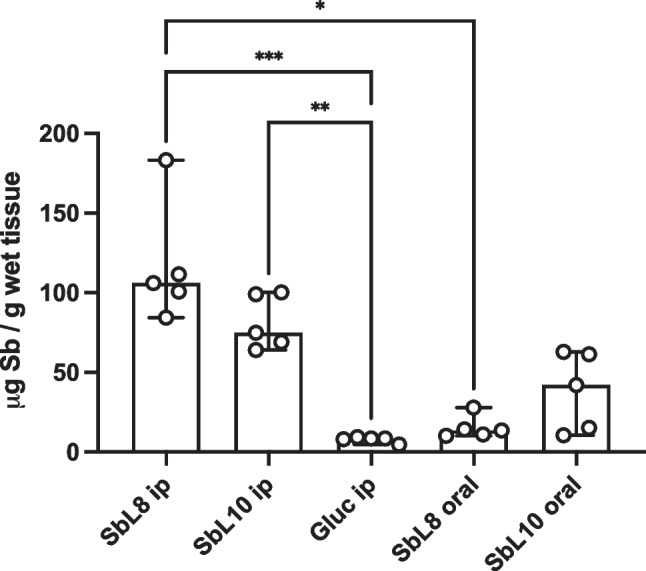
Accumulation of Sb in the liver of mice 24 h after administration of SbL8 or SbL10 by oral (200 mg Sb/kg) or i.p. (20 mg Sb/kg) route or after Glucantime® i.p. (20 mg Sb/kg). The liver was collected after 24 h, homogenized and digested with nitric acid for subsequent determination of Sb by graphite furnace atomic absorption spectroscopy. Data are shown as medians ± 95% confidence intervals (n = 5). *p < 0.05, **p < 0.01; ***p < 0.001, Kruskal–Wallis followed by Dunn’s multiple comparison test. Figure reprinted and minimally adapted from Lanza et al. (2022) in accordance with Creative Commons Attribution 4.0 International

Evaluation of the extent of Sb accumulation in the liver of mice 24 h after parenteral administration showed tenfold higher level of Sb from the amphiphilic complexes compared to Glucantime® given at the same dose (Lanza et al. 2022), as illustrated in Fig. 6.

These data evidenced an effective targeting of Sb to the liver. Opsonization of nanoparticles that consists in the surface adsorption of opsonins, such as immunoglobulins and complement proteins (i.e. C3, C4, and C5) (Moghimi and Patel 1998), may also contribute in vivo to the rapid blood clearance of SbL8 and SbL10 nanoassemblies by macrophages of the mononuclear phagocytic system. In accordance with the higher targeting of the liver, SbL8 and SbL10 also showed high antileishmanial efficacy following parenteral administration at 20 mg/kg/day in a murine model of VL, with marked reduction of parasite loads both in the liver and the spleen (Lanza et al. 2022).

A remarkable property of the SbL8 and SbL10 nanoassemblies is their high kinetic stability at neutral pH in aqueous solution, and their conformational change and release of incorporated lipophilic substance upon acidification. This conformational change is expected to occur in the parasitophorous vacuole of infected cells, in which parasites reside, and this may facilitate the delivery of Sb to the parasite.

## Other antimony derivatives involved in the formation of supramolecular assemblies

We describe below other antimony derivatives which were shown to promote to the formation of supramolecular assemblies, starting with those that have been studied for treatment of leishmaniasis.

### Antimony(V) oxide

Sb(V) nanohybrid hydrosol was synthesized by hydrolysis of SbCl_5_ in water forming sparingly soluble hydrated Sb(V) oxide (Sb_2_O_5_⋅nH_2_O), followed by purification through dialysis (MWCO = 14 kDa) to remove HCl. The purified solution was then treated with an aqueous solution of either NMG or panthenol and left overnight at room temperature under stirring. Thus, Sb_2_O_5_⋅nH_2_O nanoparticles were formed with size in the range of 35–45 nm (Franco et al. 2016). The NPs were found to be slightly more effective than MA in reducing the lesion size after intralesional administration in hamsters experimentally infected with *L. amazonensis*. The presence of nanoparticles in the cytoplasm of the infected macrophages was observed in all treated animals, suggesting an effective targeting of the host cell.

### Potassium antimony(V) salt

Nanoparticle phosphate-based composites (NPCs) were synthesized using a mixture of salt solutions in a controlled precipitation system. The salts included Na_4_P_2_O_7_.10H_2_O, CaCl_2_.2H_2_O, MgCl_2_.6H_2_O and KSb(OH)_6_ (Alvarenga et al. 2015). NPCs featured negative zeta potentials and mean diameters around 180 nm. In this study, the precise role of Sb(V) in the structuration of the nanocomposite has not been investigated. When tested in the intramacrophage model, both Sb(V)-containing and Sb(V)-lacking NPCs reduced the amastigote infection, but those containing Sb(V) were more effective. The authors proposed that NPCs may act as Sb nanocarrier and specifically target macrophages.

### Antimony(III) alkoxide and thiolate cages: role of pnictogen bonding in supramolecular assembling

**Fig. 7:**
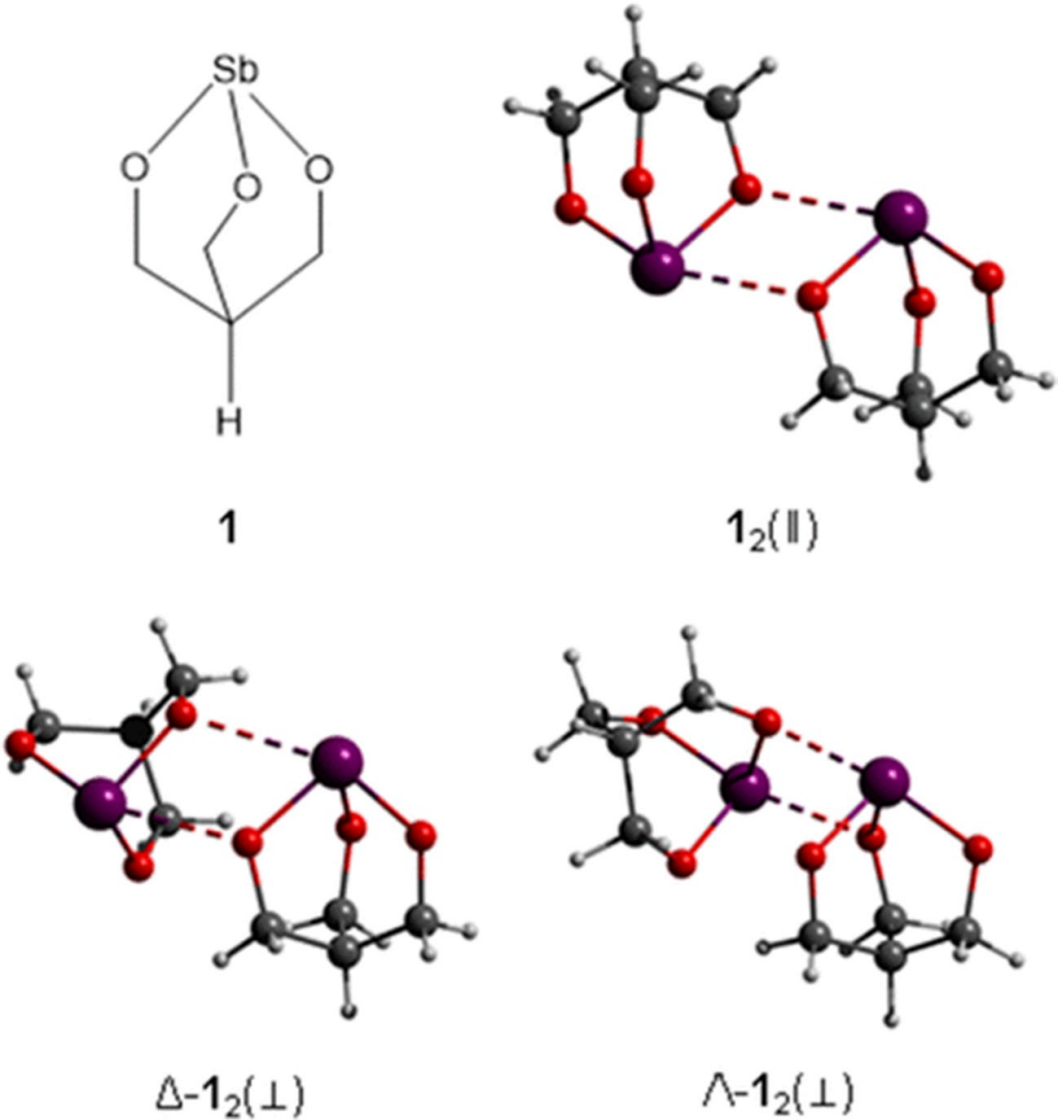
Illustration of Sb…O pnictogen bonds (dashed line) in a simple alkoxide complex. Reprinted with permission from Moaven et al. (2017), Copyright 2017 American Chemical Society

Sb(III) alkoxide and Sb(III) thiolate cages were prepared by treating a tetrahydrofuran (THF) solution of the respective alcohol or thiol with a THF solution of Sb(III) tert-butoxide, at room temperature. These antimony compounds were used as building blocks to design and construct supramolecular self-assemblies, via pnictogen bonding (Moaven et al. 2017; 2018; 2020). Pnictogen bonding consists in secondary bonding interactions between an electrophilic pnictogen atom (such as P, As, Sb and Bi) in a molecular entity (PnB donor) and a nucleophilic region in another or same molecular entity (PnB acceptor) (Mahmudov et al. 2020; Scheiner 2020; Santos et al. 2021; Varadwaj et al. 2022). These attractive forces are weaker than conventional covalent bonds (Santos et al. 2021). An example of pnictogen bonds formed from a simple Sb(III) alkoxide is shown in Fig. 7.

Interestingly, the involvement of intermolecular pnictogen bonding with these complexes led to formation of reversed bilayer vesicles. These self-assembly architectures can have up to three pnictogen bonding around the antimony center (Moaven et al. 2018; 2020). As nonaqueous environments play a dominant role in synthetic chemistry, these reversed bilayer vesicles with dynamic behavior are expected to find applications as nanocontainers or nanoreactors for carrying and delivering their cargoes in organic solutions (Moaven et al. 2020). Such supramolecular assemblies may also be incorporated into emulsified systems. Thus, it would be interesting in future works to investigate the stability of the resulting supramolecular entities and their potential as antileishmanial agent.

### Antimony(III) binding to trypanothione reductase (TR)

The Sb(III) ion was found to bind to the catalytic cleft at the dimeric interface of TR, being tetrahedrically coordinated by two cysteines, one threonine (Thr) residue and one histidine (Baiocco et al. 2009). Although not discussed by the authors, the bond between Sb and Thr may be a Sb…O pnictogen bond, as supported by the geometry of the complex and the Thr − Sb distance of 3.0 Å (Moaven et al. 2017). The formation of the TR-Sb complex is highly relevant to the mechanism of action of antimonial drugs and provides another example of supramolecular assembly stabilized by the Sb ion.

## Possible role of pnictogen bonding in the supramolecular assemblies of antileishmanial antimony(V) complexes

Although less documented and explored, Sb(V) was also found to behave as a pnictogen bond donor center (Moaven et al. 2019; Mahmudov et al. 2020). This led us to postulate that multiple pnictogen bonds, for instance Sb…O and Sb…N, may contribute to self-association in MA, MA-β-CD, SbL8 and SbL10. However, such interactions should take place when the antimonial compounds do not have a negative charge. Sb(V) complexes without negative charge are encountered when the Sb ion is pentacoordinated or the compounds are in acidic pH conditions. Accordingly, the acidification of SbL8 aqueous suspension resulted in a less negative zeta-potential of the nanoparticles (Lanza et al. 2022). This condition may favor the appearance of such secondary bonding and, in the specific case of SbL8 and SbL10, may contribute to the nanoparticle conformational change. In the case of amphiphilic Sb(V) complexes and MA, acidification was also found to enhance polydispersity (Lanza et al. 2022), supporting a higher extent of aggregation and a possible role of pnictogen bond formation. The occurrence of these interactions may also explain the slow dissociation kinetic of the high-order nanoaggregates upon dilution.

## Conclusions and perspectives

Different pentavalent antimonial compounds, including Sb(V) oxide, the first line drug Glucantime®, its complex with β-CD, Sb(V)-guanosine complex and amphiphilic Sb(V) complexes, have shown a tendency to self-assemble in aqueous solutions, forming nanoaggregates, hydrogel or micelle-like nanoparticles. Surprisingly, the resulting nanoassemblies have shown in most cases slow dissociation kinetics upon dilution and a strong influence of pH, which markedly impacted on their pharmacokinetic and therapeutic properties against leishmaniasis. Benefits include enhanced cell and tissue targeting, reduced toxicity, improved oral bioavailability, their potential use as carrier of lipophilic drug and for topical treatment. To explain these unique properties, we raised the premise that multiple pnictogen bonds could contribute to the formation of supramolecular assemblies and their dissociation kinetics. Thus, it would be important in future studies to more precisely characterize the structural organization of these nanoassemblies and investigate the occurrence of secondary bonding interactions.

Another issue that would be worth investigating in future works is whether the delivery of Sb to the host cell and the parasite through phagocytosis/endocytosis of the nanoassemblies would bypass the transporters involved in Sb resistance and keep the cell sensitive to the drug.

From a drug development perspective, administering the resulting Sb assemblies parenterally or even orally remains a challenge, because metal- and surfactant-related toxicities were observed in animal models (Frézard et al. unpublished data). On the other hand, the Sb(V)-G hydrogel, MA-β-CD and SbL8/SbL10 nanoassemblies show good promise for the topical treatment of CL, with the possibility of further incorporating, in the nanoformulation, a lipophilic antileishmanial drug. Remaining challenges to reach clinical trials include proof of concept of the therapeutic efficacy of Sb(V)-G hydrogel and MA-CD in murine model of CL and selection of the most promising nanoassemblies based on their efficacy/safety profile, in addition to drug stability and cost assessments.

Recent works in the literature evidencing that the Sb atom and pnictogen bonding can be exploited to rationally design supramolecular assemblies (Moaven et al. 2019) also opens the way to the design of new antimony(V) complexes and supramolecular assemblies for the safe and effective treatment of leishmaniases.
